# Abatacept blocks anti-citrullinated protein antibody and rheumatoid factor mediated cytokine production in human macrophages in IDO-dependent manner

**DOI:** 10.1186/s13075-018-1527-x

**Published:** 2018-02-07

**Authors:** Aline Bozec, Yubin Luo, Cecilia Engdahl, Camille Figueiredo, Holger Bang, Georg Schett

**Affiliations:** 1 Department of Internal Medicine 3 and Institute for Clinical Immunology, Friedrich-Alexander-University Erlangen-Nürnberg (FAU) and Universitätsklinikum, Ulmenweg 18, 91054 Erlangen, Germany; 2 Department of Rheumatology and Immunology, West China Hospital, Sichuan University, Chengdu, China; 3 Division of Rheumatology, Faculdade de Medicina da Universidade de São Paulo, São Paulo, Brazil; 4 ORGENTEC Diagnostika GmbH, Mainz, Germany

## Abstract

**Background:**

The anti-inflammatory effect of abatacept is most pronounced in patients with high-titer autoantibodies (including anticitrullinated protein antibodies [ACPA] and rheumatoid factor [RF]). Considering that autoantibodies trigger inflammatory cytokine production by monocytes and that abatacept binds to monocytes, influencing their functional state, we hypothesized that abatacept may effectively inhibit the production of several different cytokines by ACPA- or RF-challenged monocytes.

**Methods:**

Peripheral blood CD68^+^ monocytes stimulated with macrophage colony-stimulating factor for 24 h were exposed to random immunoglobulin G alone (negative control), purified ACPA, purified RF, or lipopolysaccharide (positive control) in cell culture plates coated with citrullinated vimentin (to allow ACPA immune complex formation). Stimulations were done in the presence or absence of abatacept or tumor necrosis factor (TNF) antibody (adalimumab) with or without indoleamine 2,3-dioxygenase (IDO) inhibitor 1-methyl-d-tryptophan. Supernatants were analyzed for key proinflammatory cytokines TNF-α, interleukin (IL)-1β, IL-6, IL-8, and chemokine (C-C motif) ligand 2 (CCL2) after 24 h.

**Results:**

Exposure to ACPA or RF significantly induced the production of TNF-α (20-fold and 27-fold, respectively), IL-1β (each 4-fold), IL-6 (12-fold and 11-fold, respectively), IL-8 (43-fold and 30-fold, respectively), and CCL2 (each 4-fold) in human monocytes. Abatacept inhibited this autoantibody-mediated upregulation of cytokines, reducing TNF-α by > 75%, IL-1β by > 65%, IL-6 and IL-8 by > 80%, and CCL2 by > 60%. In contrast, a TNF inhibitor did not influence autoantibody-induced proinflammatory cytokine production. IDO inhibition reversed the effect of abatacept and again permitted the induction of cytokine production by ACPA and RF.

**Conclusions:**

These data show that abatacept interferes with autoantibody-mediated cytokine production by monocytes through induction of IDO. This inhibitory effect on the production of several effector cytokines in RA may explain the fast anti-inflammatory effect of abatacept as well as its preferential efficacy in patients with high-titer ACPA and RF.

## Background

Rheumatoid arthritis (RA) is characterized by sustained cytokine production leading to chronic synovitis and cartilage and bone destruction [1]. The continuous supply of sufficient amounts of inflammatory mediators is crucial for maintaining the disease process in the joints of patients with RA. RA features remarkable chronicity of disease with little tendency to spontaneous resolution, which suggests that the continuous production of inflammatory cytokines outweighs physiological resolution processes [2, 3]. Macrophages are considered to play a seminal part in cytokine production in the joints of patients with RA, representing a major source of most of the prominent mediators of disease, such as tumor necrosis factor (TNF)-α and interleukin (IL)-6, but also other cytokines and chemokines involved in the disease process, such as IL-1β, IL-8, and chemokine (C-C motif) ligand 2 (CCL2) [4].

Although the causative factor for the sustained cytokine production in RA is not fully clear, autoantibodies and their immune complexes may play a central role in this process. Previous data have shown that both complexes of anticitrullinated protein antibodies (ACPA) and rheumatoid factor (RF) induce robust cytokine production from human macrophages [5–9]. This effect is mediated by the cross-linking of Fcγ receptor IIa on macrophages, representing a strong activation signal for cytokine release [6]. Newer data suggest that macrophage colony-stimulating factor (M-CSF)-primed macrophages are particularly sensitive to immune complex-mediated cytokine production, an observation that is also reflected by the local milieu in the synovial membrane of patients with RA, where large amounts of M-CSF are present [5, 10]. The leading role of autoantibodies in triggering cytokine release in patients with RA is also reflected by clinical observations which show that patients with RA with autoantibodies exhibit a more severe disease course [1].

Whether the concept of immune complex-mediated cytokine production is of relevance for the treatment of human RA is less well defined. On one hand, cytokine inhibitors such as TNF-α and IL-6R inhibitors work in both autoantibody-positive and autoantibody-negative disease, with no overt differences in the responsiveness between these subgroups of the disease [11, 12]. On the other hand, most [13, 14] but not all [15] studies have shown that the anti-inflammatory effect of the costimulation inhibitor abatacept (cytotoxic T-lymphocyte-associated antigen 4 [CTLA-4]-immunoglobulin [Ig]) is more pronounced in patients with high-titer autoantibodies (including ACPA and RF). Furthermore the anti-inflammatory effect of abatacept is remarkably fast and indistinguishable from TNF-α inhibition, indicating that the anti-inflammatory effect of this drug may be based on a blockade of antibody-mediated cytokine production by macrophages [16]. Cluster of differentiation 80 (CD80) and CD86, the receptors for abatacept, are abundantly expressed on macrophages [17], and previous data on dendritic cells and osteoclasts, which are derived from the same cell lineage as macrophages, have shown that abatacept can directly influence the function of these cells through the activation of indoleamine 2,3-dioxygenase (IDO) [18–20].

Considering that autoantibodies trigger inflammatory cytokine production by monocytes [5–8] and that abatacept binds to monocytes, influencing their functional state [21], we hypothesized that abatacept may effectively inhibit the production of several different cytokines by ACPA-or RF-challenged monocytes. We therefore (1) tested whether abatacept can inhibit the production of inflammatory cytokines by monocytes exposed to ACPA or RF, (2) compared the effect with the one obtained with TNF-α inhibition, and (3) investigated whether a potential effect of abatacept on cytokine production is based on the induction of the enzyme IDO.

## Methods

### Preparation of human monocytes

Peripheral blood mononuclear cells were isolated from 10 healthy donors and 30 patients with RA by Ficoll density gradient centrifugation. The procedure was approved the ethics committee of the University Clinic of Erlangen, and written informed consent was obtained from all healthy individuals and patients with RA. Monocytes were subsequently sorted for the common monocyte marker CD68 and suspended in DMEM supplemented with 5% FCS (Biochrom, Cambridge, UK) and 1% Gibco penicillin-streptomycin (Life Technologies, Carlsbad, CA, USA). Cultures were performed with 5 × 105 cells per well in a 24-well plate for 48 h**.** In addition, 10 ng/ml M-CSF was added to the cultures over a period of 24 h to induce differentiation of monocytes. This method has been shown to increase the responsiveness of monocytes to immune complexes [5].

### Isolation of ACPA and RF

Pooled serum samples were obtained from three patients with RA (fulfilling American College of Rheumatology/European League Against Rheumatism [ACR/EULAR] classification criteria; mean ± SD age 56 ± 12 years; two females, one male; disease duration 7.2 ± 2.3 years; Disease Activity Score in 28 joints [DAS28] 3.0 ± 0.5) with a high-titer ACPA response (> 1000 IU/ml in cyclic citrullinated peptide 2 [CCP2]; Phadia/Thermo Fisher Scientific, Uppsala, Sweden) and a high-titer RF response (> 100 IU/ml; IBL/Tecan, Morrisville, NC, USA). All three patients also showed high autoantibody reactivity against citrullinated vimentin (> 100 IU/ml; ORGENTEC Diagnostika, Mainz, Germany). Prior to affinity purification of ACPAs, immunoglobulin G (IgG) was purified by affinity chromatography on a protein A column (GE Healthcare Life Sciences, Pittsburgh, PA, USA). Eluted IgG fractions were immediately neutralized with a 1:10 volume of 1 M Tris buffer (pH 8.0) and dialyzed against PBS (pH 7.2). For isolation of ACPA, recombinant mutated and citrullinated vimentin (MCV) was conjugated to cyanogen bromide-activated sepharose (GE Healthcare Life Sciences) (1 mg protein/1 ml equilibrated sepharose) according to the manufacturer’s instructions and stored at 4 °C in PBS with 0.1% bovine serum albumin. The purified IgG fraction was applied to a separate citrullinated vimentin affinity column and incubated for 2 h at 4 °C, rotating end over end. The column was washed with 0.5 M NaCl with 0.05% Tween 20 in PBS (pH 7.2), and the unbound fraction was collected as previously described [22]. The bound ACPAs were eluted with 3.5 M MgCl_2_ (pH 7.5). The high salt buffer of the purified IgG fractions was exchanged against PBS using a Zebra Spin desalting column (Pierce Biotechnology/Thermo Fisher Scientific, Rockford, IL, USA). ACPA concentration was increased by ultrafiltration on a Vivaspin 30 K unit (MilliporeSigma, Burlington, MA, USA). The content of the MCV ACPA was verified with an enzyme-linked immunosorbent assay (ELISA) for measuring CCP2 and MCV reactivity. The ACPA preparation tested negative for RF. IgG-depleted serum was used to purify the IgM fraction by loading it onto an IgM-capturing matrix (CaptureSelect IgM Affinity Matrix; Life Technologies, Leiden, The Netherlands). The IgM RF fraction was obtained by loading onto a human IgG matrix (IgG Sepharose; GE Healthcare Life Sciences) as previously described [7]. The RF fraction was confirmed for RF-IgM reactivity after elution, but had no ACPA reactivity. Eluted fractions were neutralized by adding 2 M Tris right after collection. ACPA and RF fractions were negatively tested for lipopolysaccharide (LPS) contamination using the Pierce LAL Chromogenic Endotoxin Quantitation Kit (Thermo Fisher Scientific).

### Stimulation of macrophages to ACPA immune complexes and RF

For the exposure of monocytes to ACPA immune complexes, plates were precoated with 10 μg/ml citrullinated vimentin (ORGENTEC Diagnostika) as a target antigen for 24 h. Then monocytes were added and stimulated with M-CSF for 24 h. For immune complex formation, 100 μg/ml purified anticitrullinated vimentin antibody was added to the cultures. Supernatants were taken for cytokine analysis after 24 h of immune complex exposure. RF was also added to the cultures 24 h before the supernatants were harvested. The concentration of immune complexes was chosen according to previously published in vivo concentrations in the synovium of patients with RA [23, 24]. As a negative control, cells were treated with 100 μg/ml random IgG in a similar matter as described for ACPA and RF, and stimulation of cells with 0.5 ng/ml LPS (Sigma-Aldrich, St. Louis, MO, USA) served as a positive control.

### Assessment of cytokine and chemokine levels

Supernatants of the macrophage cultures were stored at −80 °C. After thawing, the probes were analyzed for the concentrations of TNF-α, IL-1β, IL-6, IL-8, and CCL2 using a commercial ELISA (R&D Systems, Minneapolis, MN, USA).

### Expression of IDO and nuclear factor-κB-inducing kinase

Macrophages exposed to 10 μg/ml CTLA-4-Ig for 6 h were washed twice with PBS and homogenized into extraction buffer (8 M urea, 10% glycerol, 1% sodium dodecyl sulfate [SDS], 10 mM Tris-HCl, pH 6.8, protease inhibitor cocktail [Roche Diagnostics, Indianapolis, IN, USA], and 1 mM sodium vanadate). Total cell lysates were resolved on 10% SDS-PAGE gels and transferred to nitrocellulose membrane (Bio-Rad Laboratories, Hercules, CA, USA). IDO expression was detected using antihuman IDO antibody (MilliporeSigma), nuclear factor-κB-inducing kinase (NIK) was detected by anti-NIK antibody (Cell Signaling Technology, Danvers, MA, USA), and antiactin antibody (Sigma-Aldrich) was used as a loading control. For quantifying IDO mRNA expression by RT-PCR, RNA was extracted from human macrophages exposed to 10 μg/ml CTLA-4-Ig for 1 h using TRIzol reagent (Life Technologies), and complementary DNA (cDNA) was synthesized by using a high-capacity cDNA reverse transcription kit (Thermo Fisher Scientific) according to the manufacturer’s instructions. Quantitative PCRs were performed using SYBR Green I deoxythymidine triphosphate (Eurogentec, Cologne, Germany). Specific primers for IDO were 5′-CATCTGCAAATCGTGACTAAG-3′ (foreward) and 5′-CAGTCGACACATTAACCTTCCTTC-3′ (reverse).

### Effects of abatacept on immune complex-mediated cytokine production

To analyze whether abatacept or a TNF-α inhibitor (adalimumab) affects cytokine production by monocytes exposed to immune complexes, abatacept was added to the cultures immediately before the addition of either ACPA or RF. Doses of abatacept were applied according to the therapeutic serum concentrations in humans at 10 μg/ml. Stimulations were also done in the presence of 10 μg/ml of the TNF inhibitor adalimumab. Dose dependency of the effect of abatacept and adalimumab was assessed by exposing the cultures to a large concentration range of these antibodies ranging from 1 ng/ml to 100 μg/ml (1, 10, and 100 ng/ml and 1, 10, and 100 μg/ml). Time dependency of the effect of abatacept and TNF inhibitors was assessed by adding abatacept or adalimumab either 1 h before the addition of immune complexes or simultaneously with immune complexes or 1, 3, or 6 h after addition of immune complexes.

### Mechanistic studies using IDO inhibition

For testing whether the effect of abatacept on immune complex-mediated cytokine release is dependent on IDO, experiments were performed in the presence of absence 1 mM of the IDO-specific inhibitor 1-methyl-d-tryptophan (1-MT; Sigma-Aldrich) [25]. 1-MT was added together with either abatacept or adalimumab.

### In vivo studies

Monocytes were isolated from patients with RA who fulfilled the ACR/EULAR classification criteria and were treated with either methotrexate, abatacept, or adalimumab treatment (each group, *n* =10) in the same way as described above. Characteristics of methotrexate-treated patients were as follows: age 54 ± 8 years; six females, four males; disease duration 4.1 ± 2.5 years; DAS28 score 3.1 ± 0.6. Characteristics of CTLA-4-Ig-treated patients were as follows: age 57 ± 8 years; seven females, three males; disease duration 5.0 ± 1.1 years; DAS28 score 2.9 ± 0.4. Characteristics of TNF antibody-treated patients were as follows: age 54 ± 10 years; five females, five males; disease duration 4.2 ± 3.1 years; DAS28 score 3.0 ± 0.7. The three groups were balanced with respect to age, sex, and disease duration and activity. Cells were differentiated into macrophages with M-CSF over 48 h and then exposed to 100 μg/ml purified anticitrullinated vimentin IgG. Control subjects were exposed to random IgG. Cytokine and chemokine release into supernatants were assessed as described above.

### Statistical analysis

Immune complex-mediated cytokine and chemokine levels were compared between the respective treatment groups (abatacept or adalimumab) and the control group (receiving control IgG) using the Mann-Whitney *U* test. A *p* value less than 0.05 was considered statistically significant.

## Results

### Effects of ACPA and RF on cytokine expression by macrophages

**Fig. 1:**
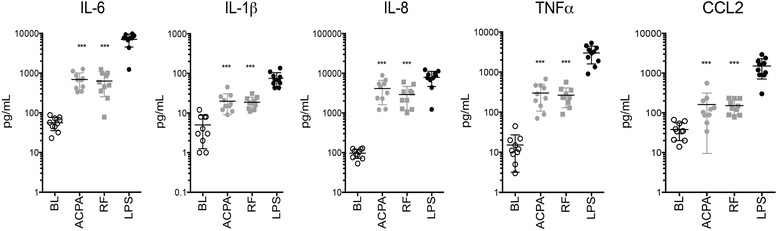
Anticitrullinated protein antibodies (ACPA)- and rheumatoid factor (RF)-induced cytokine production in monocytes. Levels of interleukin (IL)-6, IL-1β, IL-8, tumor necrosis factor (TNF)-α, and chemokine ligand 2 (CCL2) were measured in the supernatants of monocytes stimulated with 100 μg/ml ACPA immune complexes or RF or with 0.5 ng/ml lipopolysaccharide (LPS; positive control). ***p < 0.01. *BL* Baseline (exposure to random immunoglobulin G, negative control)

We first analyzed whether ACPA and RF induce cytokine expression in M-CSF-primed human monocytes. Exposure to either ACPA or RF led to a multifold increase in the production of TNF-α (20-fold and 27-fold, respectively), IL-1β (each 4-fold), IL-6 (12-fold and 11-fold, respectively), IL-8 (43-fold and 30-fold, respectively), and CCL2 (each 4-fold) by monocytes (Fig. 1). These findings support earlier investigations by revealing that both ACPA immune complexes and RF induce cytokine production by human macrophages.

### Abatacept but not TNF inhibition blocks autoantibody-mediated cytokine production

**Fig. 2:**
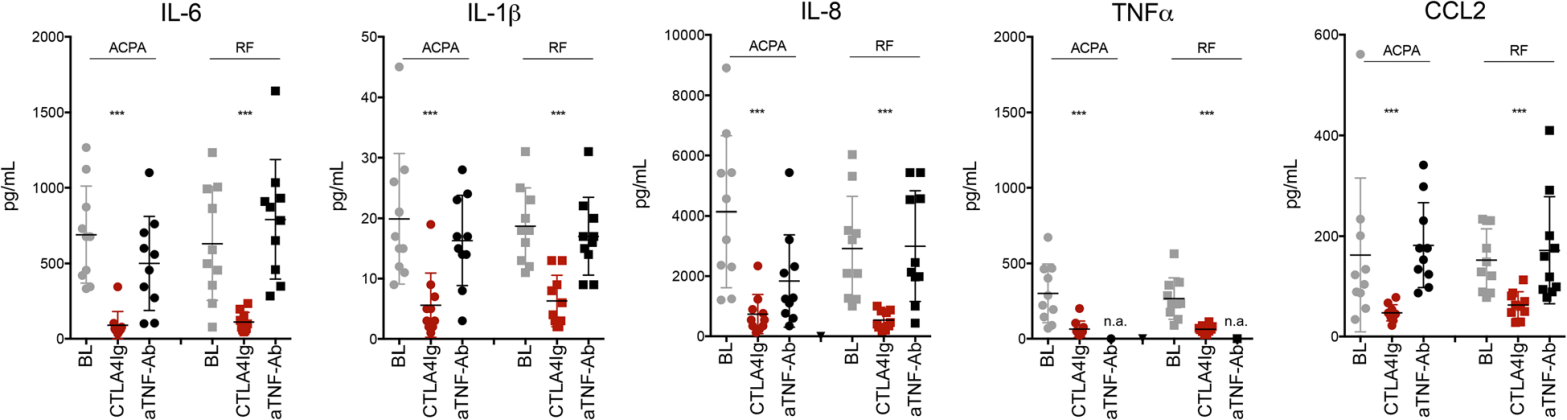
Cytotoxic T-lymphocyte-associated antigen 4 immunoglobulin (CTLA-4-Ig) blocks autoantibody-mediated cytokine production by monocytes. Levels of interleukin (IL)-6, IL-1β, IL-8, tumor necrosis factor (TNF)-α, and chemokine ligand 2 (CCL2) were measured in the supernatants of monocytes stimulated with 100 μg/ml anticitrullinated protein antibody (ACPA) immune complexes or rheumatoid factor (RF). Cultures were exposed to either 10 μg/ml CTLA-4-Ig (abatacept) or anti-TNF antibody (Ab) adalimumab. BL, baseline (neither CTLA-4-Ig/abatacept nor anti-TNF antibody/adalimumab present). ***p < 0.01

We next tested whether abatacept interferes with ACPA- and RF-mediated cytokine production. Abatacept concentrations resembling serum concentrations in patients with RA treated with abatacept significantly inhibited the production of TNF-α (−77%), IL-1β (−65%), IL-6 (−83%), IL-8 (−83%), and CCL2 (−71%) by monocytes stimulated with ACPA. Similarly, abatacept also significantly inhibited RF-induced production of TNF-α (−79%), IL-1β (−74%), IL-6 (−88%), IL-8 (−82%), and CCL2 (−60%) (Fig. 2). In contrast, TNF inhibition by adalimumab did not significantly reduce autoantibody-induced cytokine production by monocytes (Fig. 2), suggesting that the anti-inflammatory effect of TNF inhibition is based on downstream neutralization of TNF-α as an effector cytokine of inflammation. For control purposes, additional experiments were also carried out with etanercept instead of adalimumab, which also did not affect cytokine production and was indistinguishable from adalimumab (data not shown).

### Dose and time responses of abatacept-mediated effects on inflammatory cytokines

**Fig. 3:**
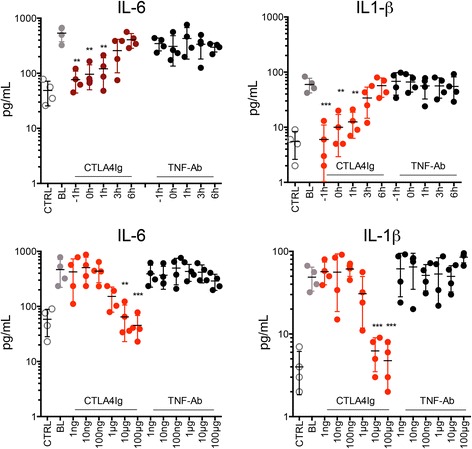
Dose and time dependence of cytotoxic T-lymphocyte-associated antigen 4 immunoglobulin (CTLA-4-Ig)-mediated blockade of autoantibody-mediated cytokine production. Levels of interleukin (IL)-6 and IL-1β were measured in the supernatants of monocytes stimulated with 100 μg/ml anticitrullinated protein antibody (ACPA) immune complexes. *Upper graphs*: Time response. Abatacept (CTLA-4-Ig; *red*) or adalimumab (tumor necrosis factor antibody [TNF-Ab]; *black*) was added either 1 h before addition of ACPA (−1 h); at the time of ACPA challenge (0 h); or 1, 3, or 6 h after ACPA challenge. *Lower graphs*: Dose response. Different doses (1 ng, 10 ng, 100 ng, 1 μg, 10 μg, and 100 μg) of abatacept or adalimumab were added to the cultures. **p < 0.05, *** *p* < 0.01

To tackle the doses of abatacept necessary to block inflammatory cytokine production elicited by ACPA and RF, we tested a wide concentration range of abatacept in these assays. Whereas concentrations below 1 μg/ml did not inhibit autoantibody-mediated IL-6 and IL-1β production, these effects started at 1 μg/ml and peaked at 10 μg/ml of abatacept with no further increase up to 100 μg/ml. Similar dose ranges of adalimumab did not affect ACPA- or RF-induced cytokine production. Regarding time response, abatacept was effective in inhibiting inflammatory cytokine production when administered before, at the time of, or up to 1 h after the exposure of the cells with either ACPA or RF. Later administration, however, did not affect cytokine production any further (Fig. 3).

### Cytokine inhibition by abatacept is mediated by IDO

**Fig. 4:**
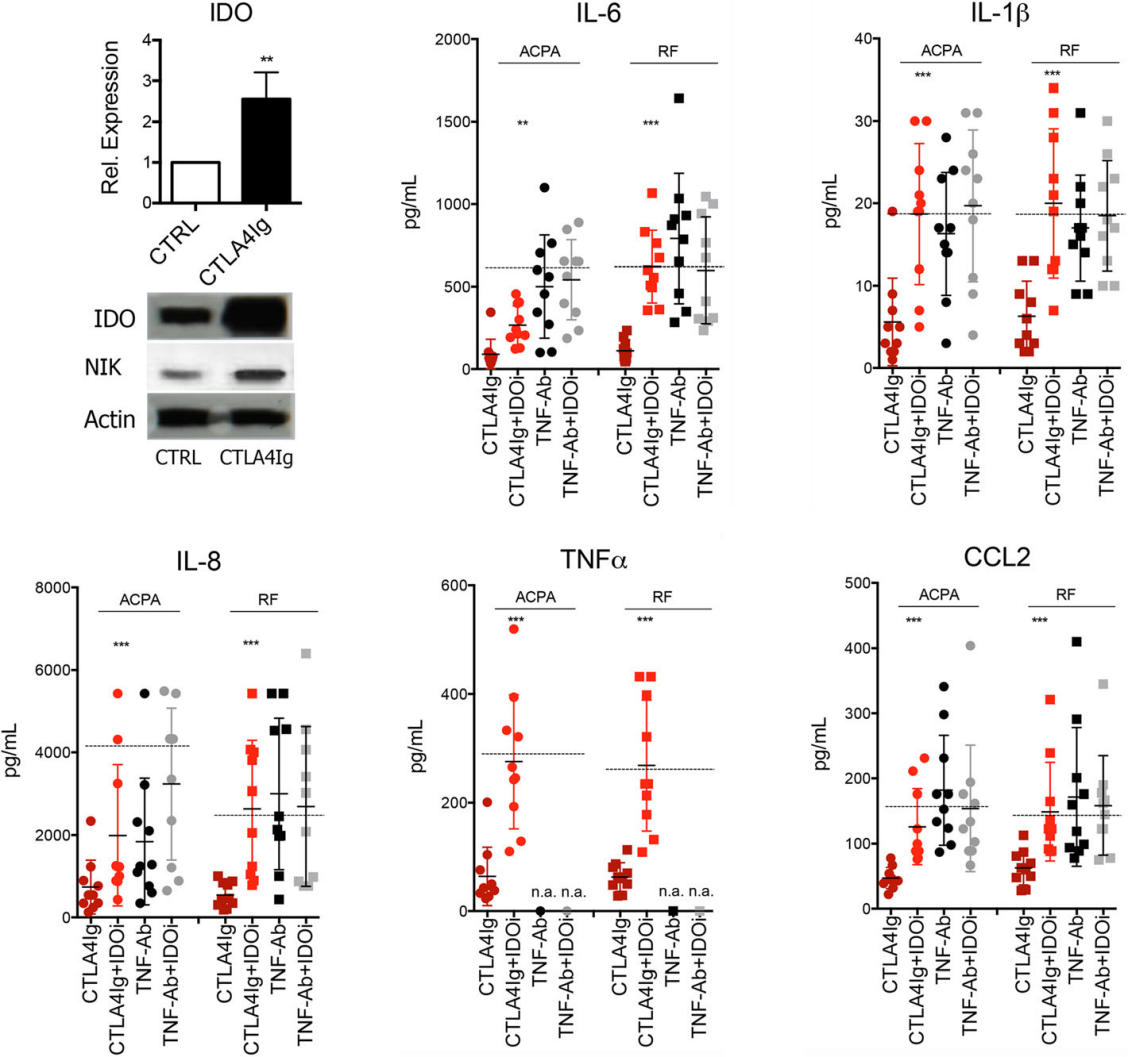
Reversal of abatacept mediated blockade of autoantibody-mediated cytokine production by indoleamine 2,3-dioxygenase (IDO). *Upper left*: RT-PCR for IDO expression in nonstimulated monocytes and monocytes stimulated with cytotoxic T-lymphocyte-associated antigen 4 immunoglobulin (CTLA-4-Ig). Western blots for IDO, nuclear factor-κB-inducing kinase (NIK), and action (loading control) in nonstimulated monocytes and monocytes stimulated with CTLA-4-Ig. Levels of interleukin (IL)-6, IL-1β, IL-8, tumor necrosis factor (TNF)-α, and chemokine ligand 2 (CCL2) were measured in the supernatants of monocytes stimulated with 100 μg/ml anticitrullinated protein antibody (ACPA) immune complexes or rheumatoid factor (RF). Cultures were exposed to either 10 μg/ml CTLA-4-Ig (abatacept) or anti-TNF antibody (TNF-Ab) adalimumab with or without 1 mM IDO inhibitor (IDOi) 1-methyl-d-tryptophan. ** *p* < 0.05, *** *p* < 0.01

On the basis of previous observations that binding of abatacept to murine monocyte lineage cells elicits the induction of IDO, we considered that the inhibition of autoantibody- induced cytokine expression by abatacept is mediated through IDO [20]. We confirmed these data in human cells by showing increases of IDO mRNA and IDO protein after treatment of human macrophages with abatacept. Also, upstream NIK expression was increased after abatacept treatment (Fig. 4). To test this concept that IDO mediates the effect of abatacept on cytokine expression in macrophages, we added the specific IDO inhibitor 1-MT to the assays described above. Addition of 1-MT completely reversed the inhibitory effect of abatacept on TNF-α, IL-1β, IL-6, IL-8, and CCL2 expression, indicating that the induction of IDO is required to mediate the inhibitory function of abatacept on ACPA- and RF-induced cytokine expression (Fig. 4).

**Fig. 5:**
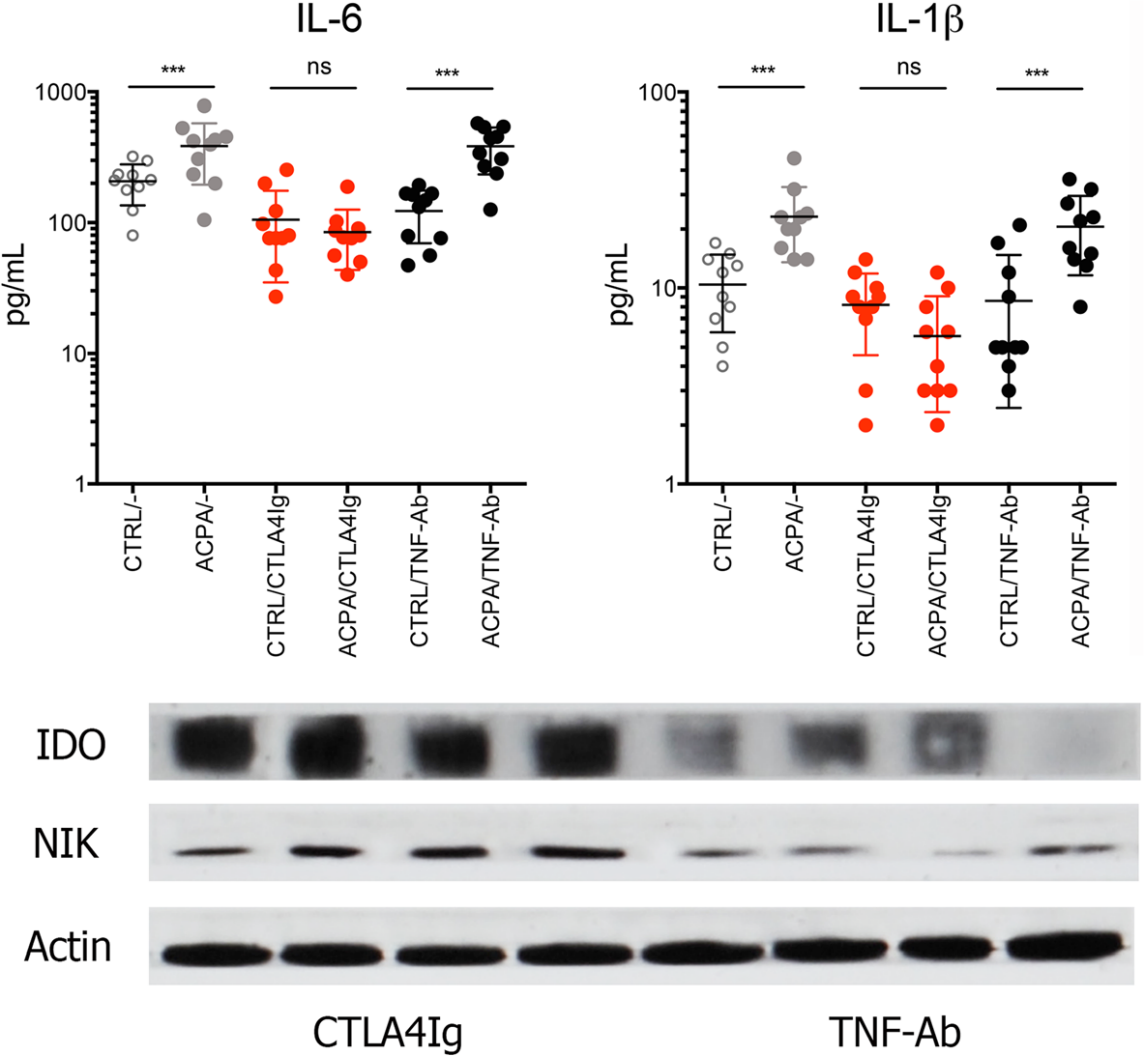
Impaired anticitrullinated protein antibody (ACPA)-induced cytokine release in monocytes isolated from abatacept-treated patients. *Upper panel*: Levels of interleukin (IL)-6 and IL-1β were measured in the supernatants of monocytes isolated from patients with rheumatoid arthritis (RA) treated with no biologic disease-modifying antirheumatic drug (“−”; only methotrexate) or cytotoxic T-lymphocyte-associated antigen 4 immunoglobulin (CTLA-4-Ig; abatacept) or tumor necrosis factor antibody (TNF-Ab; adalimumab). Monocytes were then stimulated with 100 μg/ml ACPA immune complexes or control immunoglobulin G. *** *p* < 0.001. *Lower panel*: Western blots for indoleamine 2,3-dioxygenase (IDO), nuclear factor-κB-inducing kinase (NIK), and action (loading control) expression in monocytes from patients with RA treated with abatacept (*n* = 4) or adalimumab (*n* = 4)

### Effects of abatacept on monocyte-mediated cytokine production in vivo

To test the effects of abatacept on ACPA- and RF-induced cytokine production in vivo, we isolated monocytes from patients with RA the day after they had received an injection with abatacept or adalimumab (each *n* = 10). Methotrexate-treated patients with RA served as control subjects. Cells were differentiated with M-CSF as described above and then exposed to either ACPA or RF before cytokine production was measured. In monocytes isolated from methotrexate-treated patients with RA, exposure to ACPA significantly induced the production of IL-6 and IL-1 (Fig. 5). In contrast, monocytes isolated from patients with RA treated with abatacept showed a 79% impairment of ACPA-elicited IL-6 production and a 76% impairment of IL-1 production. In contrast, no such effects on ACPA-induced IL-6 and IL-1 production were observed in patients with RA treated with the TNF inhibitor adalimumab. Finally, we were able to assess IDO expression in monocytes from each of four patients with RA treated with abatacept or adalimumab. IDO and NIK expression was higher in abatacept-treated than in adalimumab-treated patients, suggesting that abatacept induced IDO expression in human monocytes in vivo (Fig. 5).

## Discussion

Recently, the AMPLE (Abatacept versus Adalimumab Comparison in Biologic-Naïve RA Patients with Background Methotrexate) study, comparing abatacept with the TNF inhibitor adalimumab, provided conclusive evidence that abatacept leads to rapid control of signs and symptoms of inflammation in patients with RA [14]. In fact, the kinetics of onset of action of abatacept seemed to be comparable to the direct cytokine blockade, which is surprising because the primary mode of action of abatacept is considered to be the inhibition of T-cell costimulation. The rapid anti-inflammatory effects of abatacept can hardly be explained by controlling T-cell costimulation or the regulation of downstream autoantibody production. For instance, it has been shown that effects of abatacept on autoantibody levels take about 6 months before the first significant decrease of autoantibody titers is observed [26]. Furthermore, T cells likely do not constitute the main source of inflammatory cytokine production in patients with RA. An attractive explanation for the rapid anti-inflammatory activity of abatacept, however, is that binding of abatacept to CD80/86, which is highly expressed on monocytes, affects their ability to produce proinflammatory cytokines.

The second key clinical observation derived from clinical trials was that the anti-inflammatory potential of abatacept is highest in patients with high-titer autoantibodies [13]. Clinically, this finding is of substantial importance because patients with RA with high-titer autoantibodies (usually ACPA plus RF) represent the most severe form of RA with the highest risk for structural damage [27]. Given that autoantibodies, particularly in the form of immune complexes, are highly effective in inducing proinflammatory cytokine production by monocytes, abatacept’s binding to monocytes may interfere with the production of several key cytokines.

Our finding that abatacept inhibits ACPA- and RF-mediated cytokine production in monocytes provides an attractive explanation for both clinical observations—its rapid anti-inflammatory effects as well as its preferential clinical efficacy in patients with RA with high-titer autoantibodies. In fact, abatacept seems to partially uncouple autoantibodies from inflammatory cytokine production, preventing the proinflammatory function of immune complexes. Fc receptor binding of such complexes has been shown not only to effectively trigger cytokine production in vitro but also to stimulate inflammatory arthritis in mice and men [5–9, 28]. Furthermore, immune complexes have been shown to stimulate CD80 and CD86 expression, making monocyte lineage cells responsive to abatacept [29]. Downregulation of several key mediators of RA, including TNF-α, IL-1β, IL-6, IL-8, and CCL2, by abatacept may provide an advantage because several downstream pathophysiological processes, such as myeloid cell influx into the synovium, fibroblast activation, cartilage degradation, and osteoclast differentiation, are simultaneously influenced by abatacept. This effect may also explain why abatacept has been shown to provide a therapeutic advantage over TNF inhibition in the subgroup of patients with RA with high-titer antibodies [13].

Induction of IDO seems to play a central role in mediating the effects of abatacept on immune autoantibody-mediated cytokine production by monocytes. IDO inhibition partially reversed the suppressive effect of abatacept on cytokine production. Induction of IDO by binding of abatacept to myeloid cells has previously been shown to occur in dendritic cells and osteoclasts and marks the induction of regulatory pathways [17–19]. IDO induction in myeloid cells has been shown to induce regulatory dendritic cell differentiation; prohibits differentiation of osteoclast; and, as shown in this work, inhibits proinflammatory cytokine production by monocytes [18–20]. In contrast, the inhibition of IDO is a promising approach to fostering the immune response against cancer [30].

## Conclusions

In summary, these data help to better explain the antirheumatic effect of abatacept. Although abatacept is crucial for inhibition of T-cell costimulation and thereby targets the deregulation of the adaptive immunity in RA, the fast onset of action as well as the preferential effect in patients with autoantibodies appear to be based on its function to act as an “immunoblock” between the immune complex and proinflammatory cytokine production by monocytes. This dual effect of abatacept may have an impact on phase-dependent treatment of RA, Hence, in established disease, the “immunoblock” function is essential to achieving fast control of inflammation. In this case, abatacept disrupts the interaction between adaptive immune pathology (immune complexes) and the innate effector pathways leading to cytokine production. In the earliest phases of disease, however, abatacept-mediated blockade of T-cell costimulation, T follicular helper cell differentiation, plasmablast generation, and autoantibody production may be even more important [25, 31]. Studies investigating this concept, especially whether abatacept can prevent disease onset in RA, are currently underway.

## Abbreviations

Ab: Antibody
ACPA: Anticitrullinated protein antibodies
ACR/EULAR: American College of Rheumatology/European League Against Rheumatism
BL: Baseline
CCL2: Chemokine (C-C motif) ligand 2
CCP: Cyclic citrullinated peptide
CD: Cluster of differentiation
cDNA: Complementary DNA
CTLA-4: Cytotoxic T-lymphocyte-associated antigen 4
DAS28: Disease Activity Score in 28 joints
IDO: Indoleamine 2,3-dioxygenase
Ig: Immunoglobulin
IgG: Immunoglobulin G
IL: Interleukin
LPS: Lipopolysaccharide
MCV: Mutated and citrullinated vimentin
1-MT: 1-Methyl-d-tryptophan
M-CSF: Macrophage colony-stimulating factor
NIK: Nuclear factor-κB-inducing kinase
RA: Rheumatoid arthritis
RF: Rheumatoid factor
TNF: Tumor necrosis factor
